# Ablation of the Pro-Apoptotic Protein Bax Protects Mice from Glucocorticoid-Induced Bone Growth Impairment

**DOI:** 10.1371/journal.pone.0033168

**Published:** 2012-03-19

**Authors:** Farasat Zaman, Dionisios Chrysis, Kirsten Huntjens, Bengt Fadeel, Lars Sävendahl

**Affiliations:** 1 Pediatric Endocrinology Unit, Department of Women's and Children's Health, Astrid Lindgren Children's Hospital, Karolinska Institutet, Stockholm, Sweden; 2 Division of Molecular Toxicology, Institute of Environmental Medicine, Karolinska Institutet, Stockholm, Sweden; Institut Jacques Monod, France

## Abstract

Dexamethasone (Dexa) is a widely used glucocorticoid to treat inflammatory diseases; however, a multitude of undesired effects have been reported to arise from this treatment including osteoporosis, obesity, and in children decreased longitudinal bone growth. We and others have previously shown that glucocorticoids induce apoptosis in growth plate chondrocytes. Here, we hypothesized that Bax, a pro-apoptotic member of the Bcl-2 family, plays a key role in Dexa-induced chondrocyte apoptosis and bone growth impairment. Indeed, experiments in the human HCS-2/8 chondrocytic cell line demonstrated that silencing of Bax expression using small-interfering (si) RNA efficiently blocked Dexa-induced apoptosis. Furthermore, ablation of Bax in female mice protected against Dexa-induced bone growth impairment. Finally, Bax activation by Dexa was confirmed in human growth plate cartilage specimens cultured *ex vivo*. Our findings could therefore open the door for new therapeutic approaches to prevent glucocorticoid-induced bone growth impairment through specific targeting of Bax.

## Introduction

Glucocorticoids (GCs) are widely used to treat inflammatory and autoimmune conditions, including a variety of life-threatening and disabling disorders. In the UK alone, more than 250,000 people are taking systemic steroids for various conditions, and at least 10% of all children require some form of GCs treatment during childhood [1]. One of the undesired side effects of Dexamethasone (Dexa), a widely used GC, is its ability to cause growth retardation in treated children [2]. It is well known that GC treatment decreases longitudinal bone growth by inhibiting chondrocyte proliferation, hypertrophy, and cartilage matrix production [3], [4], but the exact molecular mediators behind these effects are poorly understood. Undesired apoptosis could be an additional underlying mechanism involved in GC-induced growth impairment. GCs are known to induce apoptosis and reduce cell proliferation in different cell types, including chondrocytes, muscle, bone cells, and thymocytes [5], [6], [7], [8], [9], [10], [11], [12]. Although experimental data have shown that Dexa treatment may trigger apoptosis within the growth plate [3], [4], [13], [14], [15], the molecular mechanisms of GCs-induced apoptosis and its role in bone growth impairment remain elusive.

Longitudinal bone growth is a dynamic process of proliferation, differentiation and cell death that is regulated by different hormones [16]. Previous studies have revealed that disturbed chondrocyte activity within the growth plate, due to either disruption in normal cell proliferation or unscheduled apoptosis, may lead to defective longitudinal bone growth [17]. Apoptosis, a form of programmed cell death, plays a central role in tissue homeostasis, and deregulation of apoptosis has been implicated in numerous pathological conditions [18]. In mammalian cells, two major apoptotic signaling pathways exist, including the intrinsic pathway, which is dependent on the mitochondria, and the extrinsic pathway, which is regulated through so-called death receptors on the cell surface [19], [20]. A wide range of signals can trigger the intrinsic pathway, including cellular stress, radiation, cytotoxic drugs and lack of essential growth factors, which may cause release of apoptogenic proteins, such as cytochrome c, from mitochondria [21]. These events result in the activation of a cytosolic caspase cascade, which leads to the morphological changes associated with apoptosis. The Bcl-2 family of proteins control permeabilization of the outer mitochondrial membrane and thereby regulate the activation of caspases [22]. According to the so-called rheostat model of apoptosis regulation, the relative amounts of pro-apoptotic versus pro-survival members of the Bcl-2 family are critical determinants of the intrinsic cell death pathway [23]. The pro-apoptotic Bax protein is regulated by phosphorylation in an Akt-dependent manner, and this phosphorylation event inhibits the effects of Bax on mitochondria by maintaining it in the cytoplasm [24].

We have previously reported that Dexa-induced apoptosis in chondrocytes is mediated through suppression of the PI3K-Akt signaling pathway and activation of the caspase cascade (caspases 3, 8 and 9) [5]. Although caspase-8 is often thought to be activated exclusively through the death receptor pathway, recent studies show that activation of caspase-8 can also occur in a mitochondria-dependent manner that is independent of the Fas-associated death domain (FADD) [25], [26]. Moreover, we reported that Dexa suppresses Akt-phosphorylation in proliferative chondrocytes [5]. Taken together, this led us to hypothesize that the intrinsic apoptotic pathway may play a key role in Dexa-induced apoptosis in growth plate chondrocytes. Using an array of different *in vitro* and *in vivo* model systems, we here demonstrate a functional role of Bax, a key mediator of intrinsic apoptosis, in GC-induced bone growth impairment. These results have important implications for the development of preventive strategies aiming to rescue longitudinal bone growth in patients receiving GC treatment during childhood.

## Results

### Bax deficiency protects young mice from Dexa-induced bone growth impairment

**Figure 1:**
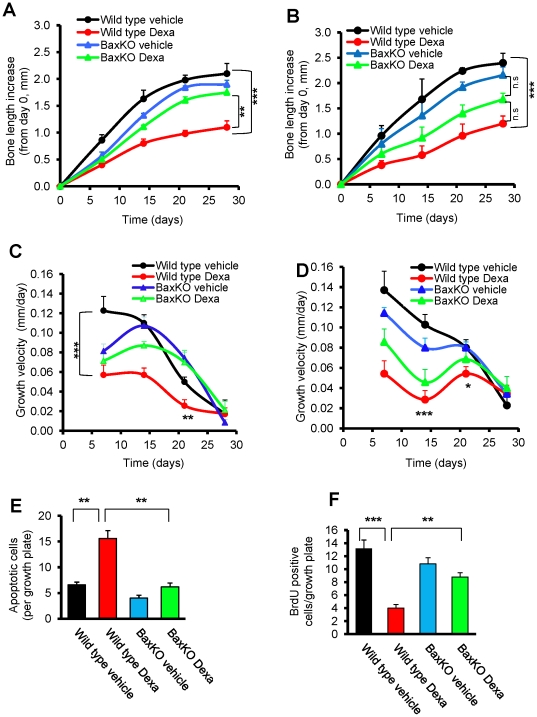
Bax deficiency protects mice from Dexa-induced bone growth impairment. Bax knockout (BaxKO) and wild-type female and male mice were treated with Dexa (2 mg/kg body weight/day) or saline for 28 days. X-ray images were captured on days 0, 7, 14, 21, and 28, and left femur lengths were measured. Femur bone length increase from day 0 in (A) female mice (***p<0.001, wild type Dexa vs. wild type vehicle; **p<0.01, wild type Dexa vs. BaxKO Dexa; *n* = 5) and (B) male mice (***p<0.001, wild type Dexa vs. wild type vehicle., n.s = not significant; *n* = 5.). Femur growth velocity (mm/day) in (C) female mice (***p<0.001, wild type Dexa vs. wild type vehicle.,**wild type Dexa vs. BaxKO Dexa., *n* = 5) and (D) male mice (***p<0.001, *p<0.05, wild type Dexa vs. wild type vehicle; *n* = 5). (E) TUNEL assays for the detection of apoptotic chondrocytes in growth plates of BaxKO and wild-type female mice treated with Dexa or vehicle for 28 days (**p<0.01, wild type Dexa vs. wild type vehicle., **p<0.01, wild type Dexa vs. BaxKO Dexa, *n* = 5). (F) BrdU incorporation analysis for the assessment of chondrocyte proliferation in growth plate cartilage of BaxKO and wild-type female mice treated with Dexa or saline for 28 days. (***p<0.001, wild type Dexa vs. wild type vehicle; **p<0.01, wild type Dexa vs. BaxKO Dexa; *n* = 5).

Based on the assumption that Bax plays a key role regulating the intrinsic apoptotic signaling pathway in chondrocytes exposed to Dexa, one would predict that Bax-deficient mice are resistant to Dexa-induced growth retardation. To validate this hypothesis, Bax knockout (BaxKO) and wild type mice (males and females) were treated with a clinically relevant dose of Dexa (2 mg/kg body weight/day) or vehicle for 28 days. In wild-type female mice, Dexa treatment reduced femur growth by 47% (p<0.001 *vs.* vehicle) while in BaxKO female mice growth was only suppressed by 8% when treated with Dexa (not significant, *vs.* vehicle; Figure 1A). The growth rescuing effect observed in Dexa treated BaxKO female mice was found to be significant when compared to wild type animals (p<0.01 *vs.* wild-type Dexa; Figure 1A). Also in male mice, Dexa caused a significant growth impairment in wild type animals (50% reduction; p<0.001 *vs.* vehicle), whereas BaxKO males were less protected from Dexa-induced growth impairment (22% reduction; p = 0.4 *vs.* females; Figure 1A–B). A similar pattern was seen when calculating femur growth velocity which was profoundly suppressed by Dexa in wild-type female and male mice, whereas Bax-deficient animals were much less affected (Figure 1C and 1D). Indeed, after 21 days BaxKO Dexa treated females showed a higher femur growth velocity than wild type females treated with Dexa (Figure 1C). Altogether, our data clearly show that BaxKO mice are protected from the negative effects of Dexa treatment on longitudinal bone growth. While the protection from Dexa-induced bone growth impairment seemed to be more pronounced in females, the difference between the two genders did not reach statistical significance.

Also body weight was affected in a similar way where BaxKO animals were less sensitive to Dexa treatment. When assessed after 4 weeks, body weight in wild type mice was 16.0±0.4 g in Dexa while 17.8±0.3 g in the vehicle group (p<0.01), a difference which was less pronounced in BaxKO female animals (16.0±0.3 g body weight in Dexa vs. 16.8±0.2 g in vehicle; not significant, p>0.2).

### Apoptosis and chondrocyte proliferation in growth plates of BaxKO mice

**Figure 2:**
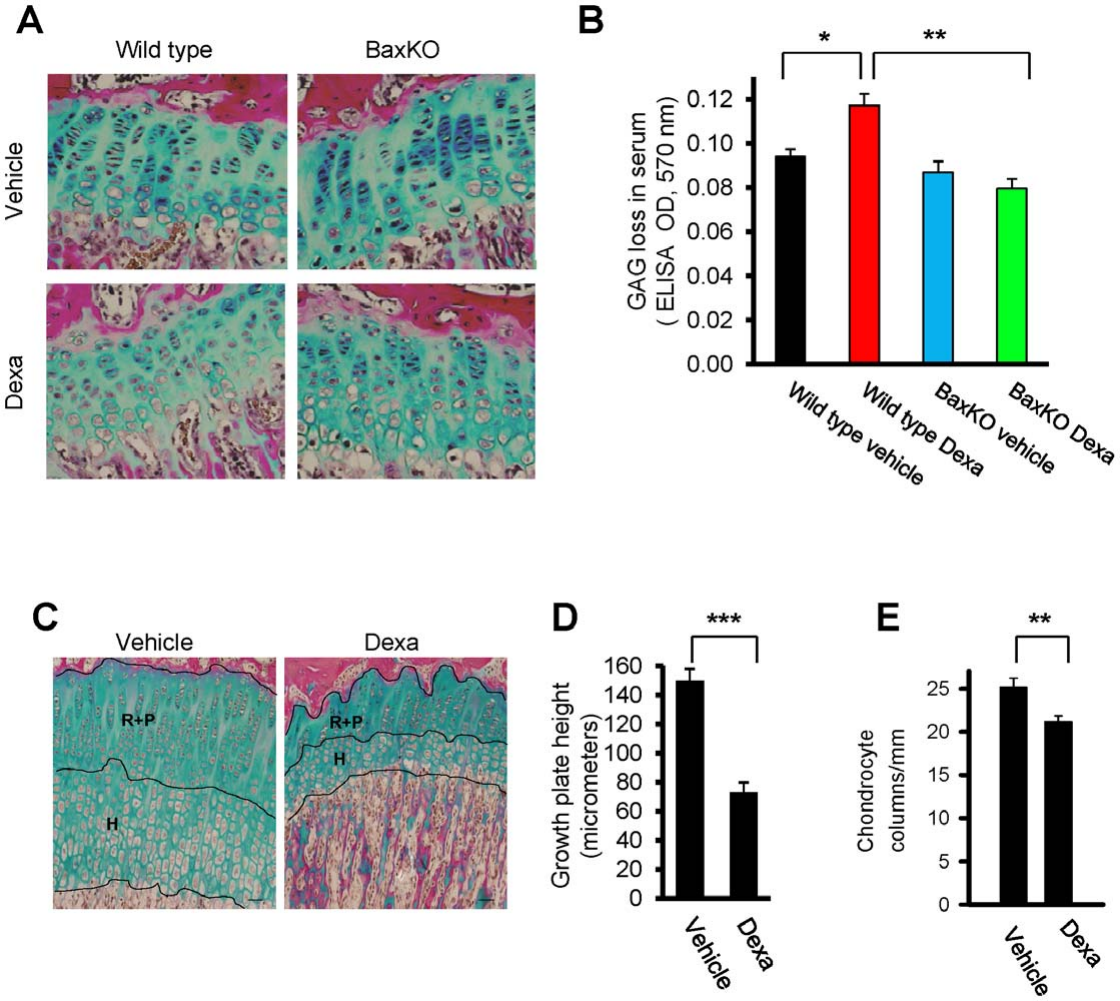
Loss of matrix and growth plate height. (A) Tibial growth plate sections from female mice were stained with Alcian Blue to detect any alterations in matrix content within the growth plate cartilage. In wild-type mice, Alcian Blue staining appeared to be weaker in Dexa-treated animals compared to those treated with vehicle, while in BaxKO no such effect was seen. Furthermore, in Dexa-treated wild-type animals growth plate height appeared to be reduced and chondrocyte columns disorganized. To quantify any changes in matrix content, (B) loss of GAG was measured in the serum demonstrating significantly increased GAG release in Dexa treated wild type mice (*p<0.05, vs. wild type vehicle., *n* = 5). In contrast, BaxKO animals were protected from Dex-induced release of GAG (**p<0.01, *vs.* wild type Dexa; *n* = 5) (C) Representative micrographs of rat growth plate (tibia) showing different zones affected by Dexa; resting+proliferative (*R*+*P*) and hypertrophic (*H*) zones (*bars* represent 50 µm). Quantitative histological analysis of (D) growth plate height and (E) chondrocyte column density (columns per mm growth plate width) in vehicle- and Dexa-treated rats (**p<0.01, ***p<0.001; *n* = 6).

The precise balance between cell proliferation and apoptosis within growth plate cartilage is a key regulator of normal bone growth. Therefore, to understand the mechanism(s) behind the protective effect of Bax-deficiency on Dexa-induced bone growth impairment, apoptosis (TUNEL staining), cell proliferation (BrdU-incorporation), matrix content (Alcian Blue staining), and morphology were studied in tibial growth plates from wild-type and BaxKO female mice. We found that Dexa treatment significantly increased the number of apoptotic chondrocytes in growth plates of wild-type mice (p<0.01; a 2.5-fold increase *vs.* vehicle; Figure 1E), while in BaxKO female mice no such effect was detected demonstrating that those animals were protected from Dexa-induced apoptosis (Figure 1E). Furthermore, Dexa treatment caused a strong suppression of chondrocyte proliferation in growth plates of wild-type mice (P<0.001) while BaxKO animals were protected from this effect (Figure 1F). Dexa treatment increased the loss of glycosaminoglycan (GAG) into the serum in wild type animals (p<0.001 *vs.* vehicle), while no effect was observed in BaxKO animals (Figure 2A–B). In addition, Dexa-treatment slightly decreased chondrocyte column density in wild-type mice (not significant), an effect which was not seen in BaxKO animals (Supplementary Information Figure *S1*). These data were validated in 7-week-old male rats treated with Dexa (5 mg/kg body weight/day) for 7 days confirming decreased tibia growth plate height (Figure 2C–D) and reduced chondrocyte column density (Figure 2E).

In summary, Bax-deficiency not only protected mice from Dexa-induced chondrocyte apoptosis but also maintained chondrocyte proliferative capacity, matrix content and growth plate integrity, which may explain how the growth of these animals is rescued during Dexa treatment.

### Bax ablation rescues chondrocytes from Dexa-induced apoptosis

**Figure 3:**
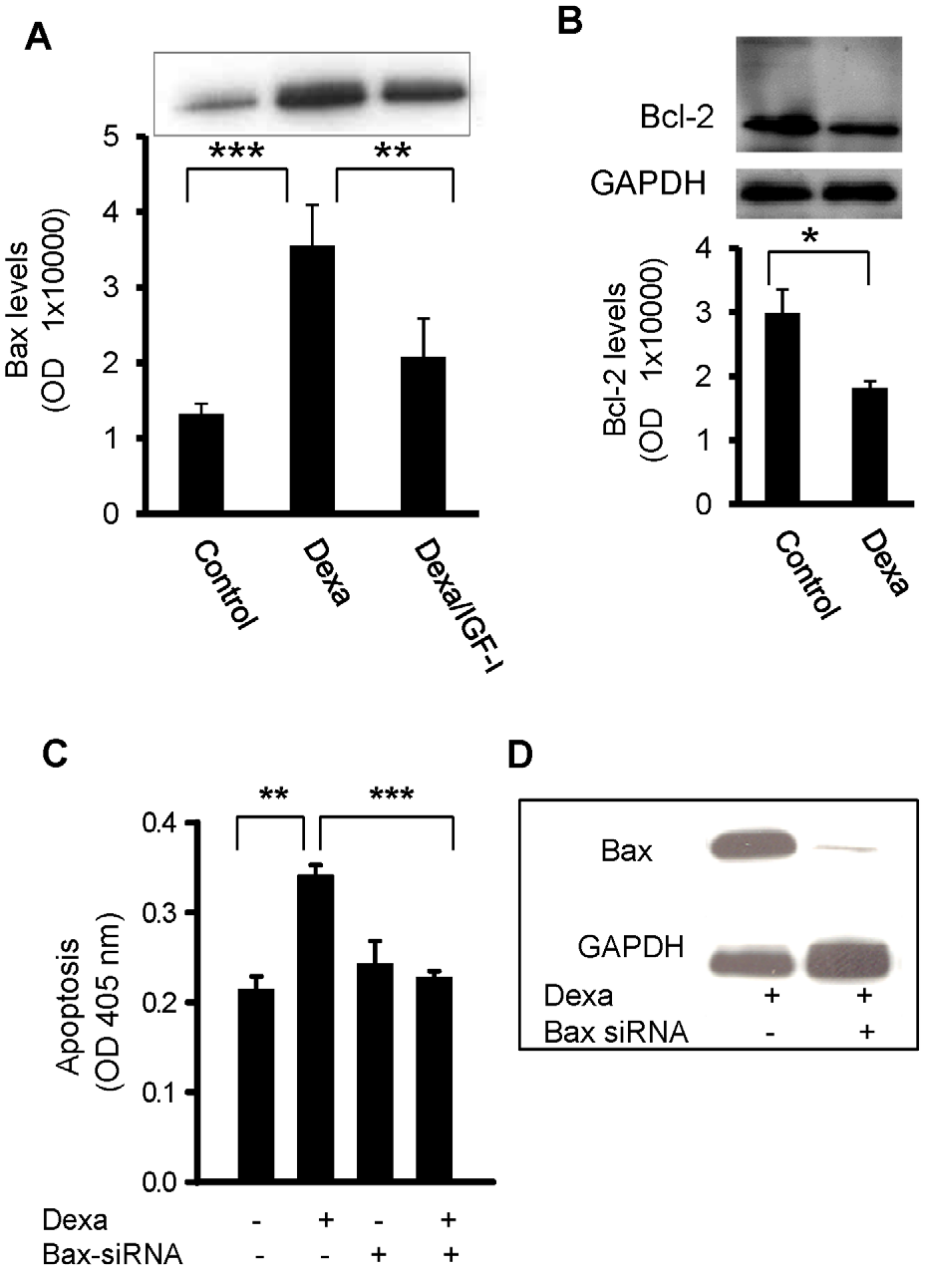
Bax ablation rescues chondrocytes. Proliferative HCS chondrocytic cells treated with Dexa (25 µM) for 72 hrs were analyzed for the expression of the (A) Bax and (B) Bcl-2 proteins. (C) Bax ablation protected proliferative chondrocytes from Dexa-induced apoptosis. HCS-2/8 chondrocytic cells in proliferative phase were transfected with Bax-specific siRNAs (80 pmol) for 48 hrs prior to incubation with Dexa (25 µmol/L) for an additional 72 hrs. Suppression of Bax completely rescued chondrocytes from Dexa-induced apoptosis. Apoptosis was measured by using the Cell Death Detection ELISA^PLUS^ kit. (D) Western immunoblotting showing suppression of Bax in Dexa-treated HCS-2/8 chondrocytic cells transfected with Bax-siRNA.

The protective effects of Bax-deficiency against Dexa-induced bone growth impairment demonstrated a critical role of this protein. To clarify any involvement of chondrocytes to mediate these effects, studies were performed in the human chondrocytic cell line, HCS-2/8. These cells exhibit characteristics of both proliferative and hypertrophic growth plate chondrocytes when kept under specific culture conditions. Experiments conducted in proliferative HCS cells revealed that 72 hrs of Dexa (25 µM) treatment markedly increased the expression of Bax (3-fold increase *vs.* control; p<0.001; Figure 3A). Co-administration of Dexa (25 µmol/L) and IGF-I (100 ng/ml) decreased Bax expression by 40% compared to Dexa alone (p<0.01; Figure 3A). Furthermore, Bcl-2 protein levels were significantly decreased in Dexa treated cells (p<0.05 Dexa *vs.* Control; Figure 3B). To investigate if up-regulation of Bax is a critical step in Dexa-induced chondrocyte apoptosis, Bax expression was suppressed using specific siRNAs. Proliferative HCS cells were transfected with Bax siRNAs and treated with Dexa (25 µmol/L) for 72 hrs. In non-silenced, control cells, Dexa increased apoptosis by 59% (p<0.001 *vs.* control); however, transfection with Bax siRNAs completely rescued chondrocytes from Dexa-induced apoptosis (p<0.001 *vs.* Dexa alone; Figure 3C), an effect similar to what was observed in BaxKO animals. Bax-specific siRNA completely suppressed the expression of Bax as shown in Figure 3D. These data suggest that Bax-deficiency in chondrocytes may help to protect the growth plate cartilage and longitudinal bone growth from the negative effects of GCs.

### Dexa treatment induces conformational changes in Bax

**Figure 4:**
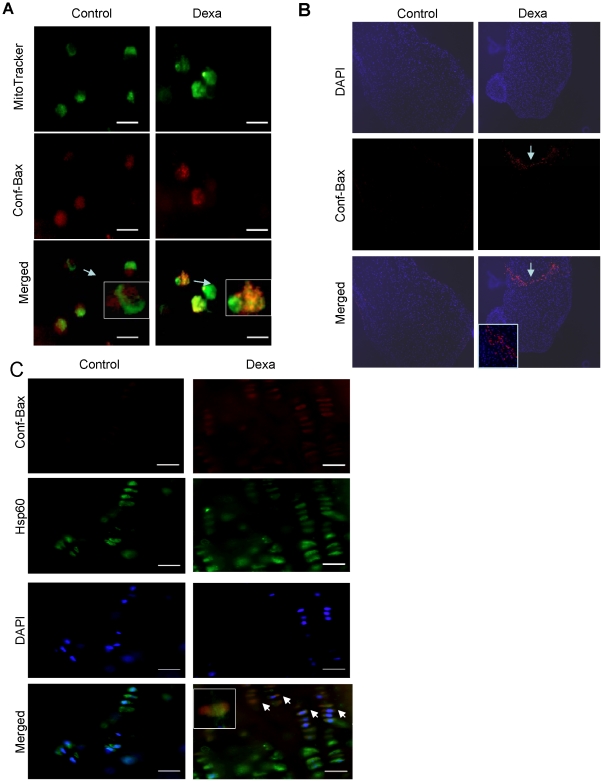
Conformational changes in Bax and translocation to mitochondria. (A) Immunocytochemistry of HCS cells in proliferation phase, treated or untreated with Dexa (25 µM) for 72 hrs. Cells were analyzed for conformational changes in Bax using a specific antibody that only detects conformationally altered Bax. Chondrocytes were labeled with Bax antibody (red) and MitoTracker (green). Bax was mainly found in the mitochondria of Dexa-treated cells, as shown by the yellow-orange staining that is due to the merged red and green fluorescence. (B) Cultured fetal rat metatarsal bones treated with Dexa stained for DAPI (blue) and conformationally altered Bax (Conf- Bax; red). (C) Growth plate sections (tibia) of 7-week-old rats treated with Dexa (5 mg/kg/day) for 7 days. Immunohistochemistry was performed by triple-fluorescent labeling for conformationally changed Bax (Conf- Bax; red), HSP60 antibody (green), and DAPI (nuclei; blue). Conformationally changed Bax was mainly found in the chondrocyte mitochondria of those animals treated with Dexa, as shown by the yellow-orange staining that is due to the merging of red and green fluorescence.

In other cell systems, conformational changes in Bax, and its resulting translocation to mitochondria, have been reported to be associated with a decrease in mitochondrial membrane potential [15]. However, in chondrocytes it is still unknown whether GC treatment can induce conformational changes in Bax. To address this key question, we used a specific antibody that detects only conformationally changed Bax (clone 6A7). HCS cells treated with Dexa for 72 hrs displayed strong mitochondrial Bax staining supporting translocation of Bax towards the mitochondrial membrane (Figure 4A). We further asked whether the observed Bax activation is due to local/direct effects of GC treatment in the growth plate cartilage. To address this, we applied an organ culture system of fetal rat metatarsal bones and treated them with Dexa (1 µmol/L) for 12 days. Bones exposed to Dexa displayed pronounced growth retardation (Figure *S2*) and increased Bax expression in growth plate chondrocytes, as determined by fluorescent immunohistochemistry (Figure 4B). To complement these *in vitro* studies, rats were treated with Dexa documenting a strong mitochondrial Bax staining in tibial growth plate chondrocytes which contrasted to vehicle treated controls where no staining was observed (Figure 4C).

### Mitochondrial membrane potential and cytochrome c release in chondrocytes

**Figure 5:**
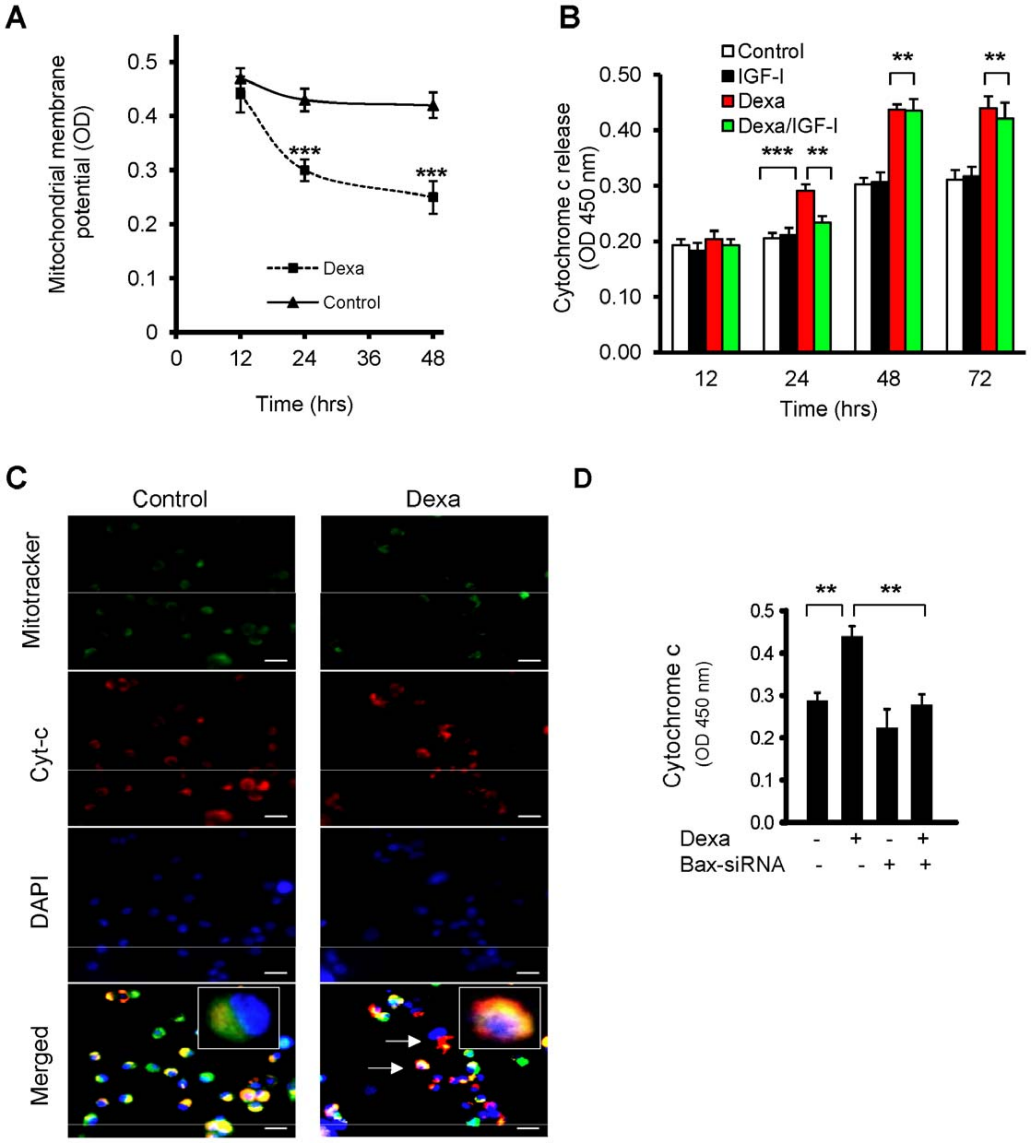
Dexa triggers dissipation of mitochondrial membrane potential and cytochrome c release in human proliferative chondrocytes. (A) Proliferative HCS-2/8 chondrocytic cells were treated with Dexa (25 µM) for 12, 24 and 48 hrs. Mitochondrial membrane potential was significantly decreased after 24 and 48 hrs in those chondrocytes treated with Dexa (***p<0.001 *vs.* untreated control). (B) HCS cells (treated or untreated with Dexa) were fractionated into cytosolic and mitochondrial extracts, and cytochrome c levels were determined in the cytosolic fractions. Cytochrome c was significantly increased following treatment with Dexa (25 µM) when assessed after 24, 48 and 72 hrs (***p<0.001 *vs.* untreated control). IGF-I (100 ng/ml) prevented Dexa-induced cytochrome c release at 24 hrs (**p<0.01 *vs.* Dexa alone), but failed to do so after 48 and 72 hrs. (C) Immunohistochemistry of cytochrome c release from mitochondria of HCS cells that were treated for 72 hrs with 25 µM Dexa or vehicle (99% EtOH). Cells were stained to visualize mitochondria (MitoTracker; *green*), cytochrome c (*red*), and nuclei (DAPI; *blue*). Cytochrome c was mainly found to be localized in the mitochondria (merged image-*yellow*) of control cells, while cytochrome c was evident in the cytosol of Dexa-treated HCS cells (shown as red fluorescence (*arrows*)). (D) Cytochrome c release was completely blocked by Bax siRNAs in transfected HCS-2/8 cells (**p<0.01; *n = 4*).

After identifying the conformational changes in Bax and its subsequent translocation to mitochondria, we measured changes in the mitochondrial membrane potential in chondrocytic HCS cells. As seen in Figure 5A, the mitochondrial membrane potential was significantly suppressed after 24 and 48 hrs of Dexa exposure (p<0.001 *vs.* control). To address if Dexa treatment also leads to mitochondrial release of cytochrome c into the cytosol, HCS cells were treated with Dexa for 24, 48 and 72 hrs and fractionated into mitochondrial and cytosolic extracts followed by quantification of cytochrome c. Dexa treatment increased cytochrome c release at 24 and 48 hrs by 45% and 43%, respectively (p<0.01 *vs.* control), and co-treatment with a known anti-apoptotic growth factor, insulin-like growth factor-I (IGF-I; 100 ng/ml), partially prevented this (21% reduction compared to Dexa alone at 24 hr; p<0.01 *vs.* Dexa alone) (Figure 5B). Cytochrome c release was also confirmed by immunocytochemistry of treated cells (Figure 5C). In untreated cells, cytochrome c was largely localized in the mitochondria, while in Dexa-treated cells cytochrome c was mainly localized in the cytoplasm. To quantify cytochrome c release, Dexa-treated chondrocytes were fractionated into mitochondrial and cytosolic extracts and measured by ELISA. Dexa alone increased cytosolic cytochrome c by 57% (p<0.01 *vs.* control) whereas Bax ablation with siRNAs completely prevented Dexa-induced release of cytochrome c (p<0.01 *vs.* Dexa-treated cells) (Figure 5D). These data suggest that Bax is a key player involved in the modulation of cytochrome c release which in turn triggers intrinsic apoptosis in chondrocytes.

### Bax activation in human growth plate cartilage biopsies

**Figure 6:**
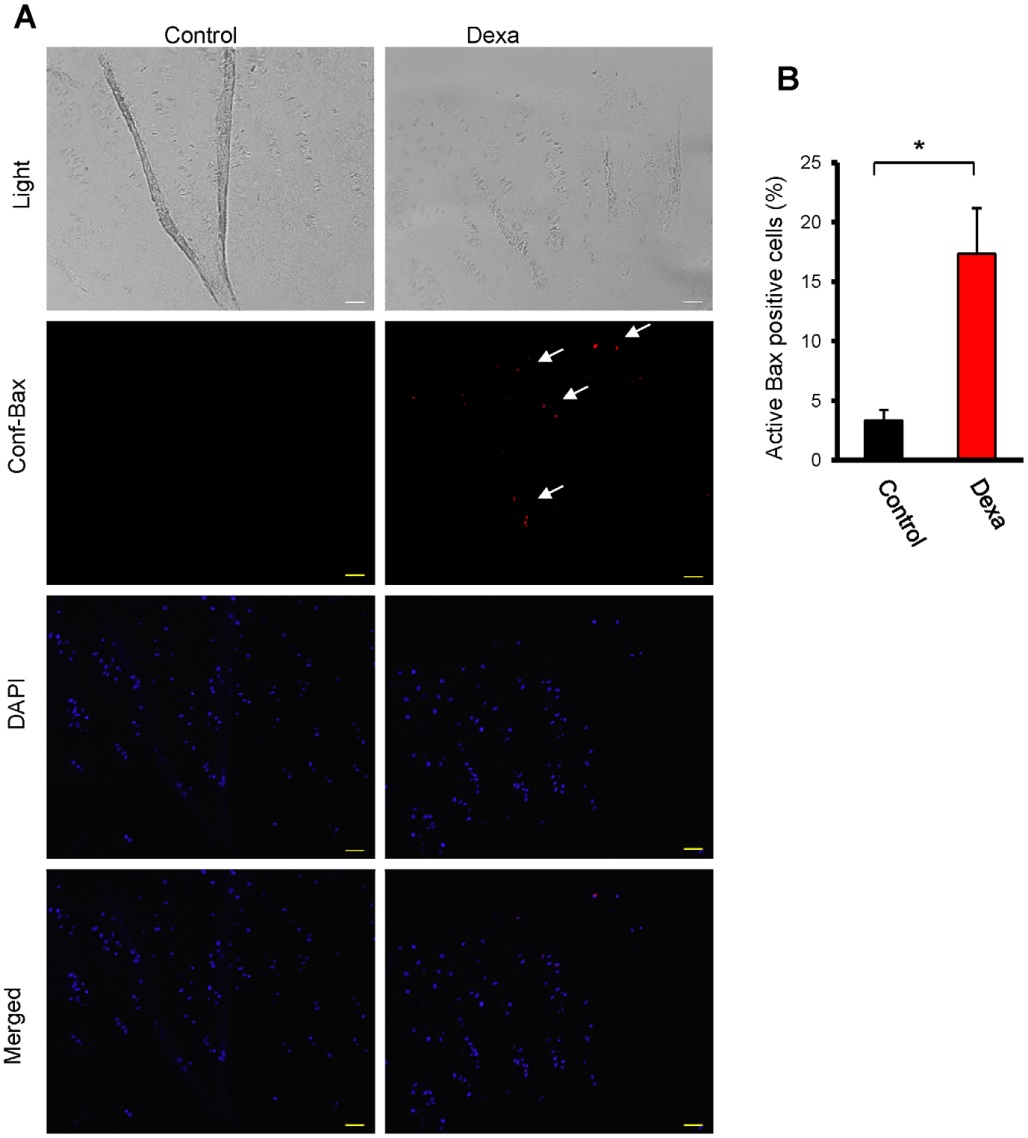
Activation of Bax in human growth plate cartilage. Human growth plate biopsies from children undergoing epiphyseal surgery to reduce their longitudinal bone growth were treated with Dexa (1 µmol/L) and vehicle (99% EtOH) for 24 hrs. (A) Immunohistochemistry for the detection of conformationally altered active Bax was performed using a specific anti-Bax antibody (clone 6A7). (B) Quantification of percent cells staining positive for conformationally altered Bax (*p<0.05, *n* = 4).

Finally, to corroborate the findings obtained in mice *in vivo*, in organ cultures of fetal rat metatarsal bones, and in a human chondrocytic cell line, studies were performed in cultured human growth plate cartilage. Growth plate tissue biopsies collected from tall children (13–15 years old) undergoing epiphyseal knee surgery to stop their continued leg growth, were treated with Dexa (1 µmol/L) for 24 hrs. Applying a Bax antibody that detects conformationally changed Bax, we observed increased Bax activation in Dexa-treated human growth plate cartilage (p<0.01 *vs.* control; Figure 6A–B). These data support Bax involvement in Dexa-induced bone growth impairment also in humans.

## Discussion

Here, we provide *in vivo* evidence that apoptosis in growth plate chondrocytes plays a significant role in GC-induced growth retardation. In addition, we show that the pro-apoptotic protein, Bax is a key player in this undesired apoptosis. The disorganization of growth plate morphology normally seen in Dexa-treated animals did not occur in Bax-deficient animals. Bax deficiency preserved chondrogenesis and rescued bones from Dexa-induced growth impairment.

We present evidence that Bax deficiency protects the growth plate from many of the undesired effects of GCs. Most striking is the strong bone growth rescuing effect observed in Dexa-treated female Bax-deficient/BaxKO mice. Longitudinal bone growth was severely impaired in Dexa-treated female wild-type mice while Bax-deficient females were protected. These findings are in accord with the previous report by Perez et al. [27] describing the alleviation of age-related bone loss in female mice following inactivation of the Bax gene [27]. Interestingly, Dexa treated Bax-deficient female animals showed a pattern of late catch-up growth while still being exposed to Dexa and eventually displayed a growth velocity which even exceeded that observed in vehicle treated Bax-deficient females, suggesting that the groups might even reach a comparable size if followed for a longer time. When compared to females, we observed that Bax-deficient male mice were less protected against GC-induced bone growth impairment. The observed sex difference could be due to the fact that infertility and testicular atrophy have been reported in Bax-deficient male mice [28]. Androgens have indeed been shown to modulate bone growth and bone mass in rats [29], and in men testicular atrophy has been linked to osteoporosis [30].

While Dexa treatment of wild-type female mice markedly increased chondrocyte apoptosis, decreased cell proliferation, and suppressed matrix production in the growth plate, no such effects were observed in Bax-deficient females exposed to Dexa. All these effects may contribute to the growth rescuing effect observed in Dexa-treated Bax-deficient female animals. However, we believe apoptosis prevention to be the most important mechanism as Bax-deficiency primarily impairs the intrinsic apoptotic pathway [22]. Furthermore, chondrocyte proliferation rate was found to be rather slower in vehicle-treated Bax-deficient female mice compared to wild-type. This suggests that cell proliferation is maintained in Dexa treated Bax-deficient mice and that chondrocytes do not undergo cell death to the same degree as in wild-type animals exposed to Dexa. Furthermore, loss of GAG into the serum, a sign of matrix degradation, was increased in wild type Dexa animals but not affected in Bax-deficient Dexa animals. We found intense Alcian Blue staining in the matrix of BaxKO animals which is likely due to higher numbers of surviving and proliferating chondrocytes producing matrix in the growth plates of those animals. Previous studies showed that under *in vitro* conditions in ATDC-5 chondrocytes, protein content increased with increase in cell number as well as extracellular matrix, whereas Dexa decreased the protein content of cultures while cell proliferation was inhibited [31]. In contrast, GCs at low doses have also been reported to prevent the loss of GAG caused by TNF-α in cultured cartilage explants [32]. Taken together, the dual role of GCs on matrix content in bone tissue appears to be time- and dose-dependent.

Applying different model systems, we demonstrate that Dexa activates the mitochondrial intrinsic apoptotic pathway in growth plate chondrocytes triggering Bax translocation and cytochrome c release in these cells. These actions of Dexa have not been described previously in chondrocytes, although in other cell model systems translocation of Bax to mitochondria is known to be an important step in the release of cytochrome c from mitochondria to the cytosol [22]. Cytochrome c in turn is an important co-factor for activation of caspases that act downstream of mitochondria c in response to apoptotic signals [18]. We and others have previously reported that increased apoptosis and altered cell proliferation/differentiation contribute to Dexa-induced bone growth impairment [5],[13]. Proliferative chondrocytes in the growth plate cartilage play a crucial role in the longitudinal bone growth process as these cells proliferate and differentiate into hypertrophic chondrocytes where their intracellular volume is dramatically increased [33]. Any disturbance in the cellular activity of proliferative chondrocytes may therefore have great impact on bone growth. We therefore decided to use proliferative chondrocytes (human HCS-2/8 chondrocytic cells) when dissecting the cellular mechanisms of Dexa-induced bone growth impairment. *In vivo* experiments were performed in rats and young mice, and *ex vivo* studies in organ cultures of fetal rat metatarsal bones and unique human growth plate biopsies. These complementary studies consistently confirmed that Bax undergoes conformational changes in growth plate chondrocytes when Dexa treated, clearly indicating that Bax plays a key role to trigger undesired growth plate chondrocyte apoptosis. Our conclusions are further supported by previous data from other model systems, including osteoblasts and mouse neural precursor cells, in which Bax has been demonstrated to undergo conformational changes upon activation, thereby triggering apoptosis [34], [35]. We have reported previously that Dexa treatment leads to suppression of Akt phosphorylation in HCS cells [5]. In other model systems such as neutrophils, Bax has been reported to be regulated by phosphorylation of serine184 in an Akt-dependent manner [24]. Phosphorylation inhibits the effects of Bax on mitochondria by maintaining Bax in the cytoplasm [24]. It is therefore likely that Dexa-induced activation of Bax reported herein is associated with suppressed Akt phosphorylation.

The present study provides strong evidence based on multiple approaches that Bax plays a major role in Dexa-induced apoptosis in chondrocytes. Moreover, Espina et al. [36] reported that gene silencing of Bax or the upstream BH3-only protein Bim both reduced apoptosis induced by Dexa in murine osteoblasts. However, we cannot rule out the existence of other minor pathways leading to apoptosis not involving Bax. We previously reported that Dexa triggers caspase-dependent apoptosis in the HCS-2/8 chondrocytic cell line with early activation of caspase-8 [5] and caspase-8 could, in principle, activate downstream effector caspases including caspase-3 directly without engagement of the mitochondrial amplification loop [18]. Notwithstanding, Bax appears to be a key player, as evidenced by the significant protection seen in BaxKO animals.

The present observation that Bax-deficient mice are resistant to Dexa-induced bone growth impairment is of considerable clinical relevance and could have wider implications. For example, cartilage tissue specimens from rheumatoid arthritis patients have higher levels of Bax than in samples from healthy controls [37]. The significance of our findings for bone physiology is further supported by previous mouse studies showing that Bax deficiency is associated with increased bone mineral density (BMD) [27] and increased cartilage production [38]. These observations suggest that decreased levels of Bax may provide further protection to the bone tissue against GCs toxicity. We propose that future treatment strategies based on targeted suppression of Bax should be explored in disease specific conditions such as chronic inflammation where GCs are widely used. Use of small molecules/peptides with anti-Bax activity such as humanin [39], [40], can be one feasible approach to block negative effects of GCs in normal tissues [41]. Interestingly, Bax knockout animals do not spontaneously develop tumors or any other health complications, except for the fact that the males are infertile [27].

In conclusion, we have shown that Dexa activates the pro-apoptotic protein Bax in growth plate chondrocytes and that Bax plays a key role in the pathogenesis of Dexa-induced bone growth impairment by triggering apoptosis and premature loss of growth plate chondrocytes. We also found Bax-deficient mice to be significantly protected from Dexa-induced chondrocyte apoptosis and bone growth impairment further supporting a crucial role of Bax in mediating these undesired effects of Dexa. In unique human growth plate tissue samples, we confirmed Bax-activation after treatment with Dexa emphasizing the importance of Bax as a key regulator of longitudinal bone growth. Our data suggest that therapeutic targeting of Bax may offer a potential strategy to prevent GC-induced bone growth impairment in treated children.

## Materials and Methods

### Ethics statement

The human growth plate tissue collection was approved by the local human ethics committee (Karolinska Institutet Research Ethics Committee North at the Karolinska Hospital). According to this approval, verbal informed consent was obtained from each subject and his/her parents which was documented in the original hospital records.

Animal studies were approved by the local animal ethics committee (Stockholm North Animal Ethics Committee, permits number Dnr N74/08, N389/07 and N29/08).

### Human growth plate biopsies

Human growth plate biopsies were collected from the tibial growth plates of four tall boys (13–15 years old) who underwent epiphyseal surgery to arrest longitudinal bone growth. Immediately after collection, the biopsies were cut into small pieces and transferred into a DMEM-high glucose 1× medium (cat # 21063, Invitrogen, Scotland) with the same additives as the medium used for metatarsal bone cultures. Biopsies were treated with Dexa (1 µM) or vehicle (99% EtOH) for 24 hrs, fixed in 4% formaldehyde, decalcified in 10% EDTS, and embedded in paraffin blocks.

### Maintenance and treatment of Bax-deficient mice

Heterozygous, Bax-deficient C57BL/6 mice [28] were obtained from Jackson Laboratories (Bar Harbor, ME), and breeding was performed to obtain homozygous animals. Genotyping was done as described by Jackson Laboratories, and homozygous (male and female) 30–32 day-old mice received a daily subcutaneous injection (in the neck) with vehicle (0.1 ml saline) or Dexa (2 mg/kg body weight) for 28 days. The mice were weighed, and bones were measured every week by X-ray (AMX-4, GE Medical Systems, WI). All animals received an intraperitoneal injection with BrdU (10 mg/ml; 200 µl), 15 minutes and 2 hrs before sacrifice. The femur and tibia were carefully dissected, cleared from muscle and immediately fixed in 4% formalin for 48 hr at 4°C. Four sections were mounted on each slide, one from each group, so that all samples were treated under the same conditions.

### Measurement of sGAG in serum

To measure the loss of matrix content in serum, we used sulphated glycosaminoglycans (sGAG)-kit (Euro-Diagnostica AB, Malmö, Sweden), a quantitative dye-binding assay for the *in vitro* analysis of sGAG. This assay can be used to detect sGAGs in biological samples such as synovial fluid, blood, and tissue extracts. The amount of proteoglycans can be reliably and conveniently quantitated with the Alcian Blue method which is highly specific for the sulfated glycosaminoglycan chains on the proteoglycans. Serum samples from both wild type and BaxKO animals were analyzed for loss of sGAG according to instructions provided by the manufacturer with the kit.

### Reagents

Dexa and IGF-I (Sigma-Aldrich, Steinheim, Germany) were dissolved in ethanol or saline, according to the manufacturer's instructions. Trypsin, phosphate buffered saline (PBS), fetal bovine serum (FBS), EDTA, MEM alpha medium, and DMEM/F12 medium were all purchased from Invitrogen (Paisley, Scotland).

### Cell culture and treatment

The human chondrosarcoma-derived chondrocytic cell line, HCS-2/8 [42], was maintained in D-MEM/F12 medium supplemented with 20% FBS, as previously described [5].

### Immunoblotting

Following incubation, HCS-2/8 cells were lysed in RIPA buffer and protein levels were measured using the Bradford assay (Bio-Rad Laboratories AB, Sundbyberg, Sweden). Cell extracts were subjected to SDS-PAGE, electrophoretically transferred to nitrocellulose membranes and subsequently incubated with primary antibodies against Bax (1∶1000), Bcl-2 (1∶1000) (Santa Cruz Biotechnology, Santa Cruz, CA). The membranes were incubated overnight at 4°C, washed with 1× TBS-T and incubated with HRP-conjugated secondary antibodies. The signals were detected using the ECLplus chemiluminescence detection system. Bax expression in HCS-2/8 cells was determined using specific anti-Bax antibodies (Santa Cruz Biotechnology, Santa Cruz, CA).

### DNA fragmentation analyses

The detection and quantification of cytoplasmic, histone-associated DNA fragments (Cell Death Detection ELISA^PLUS^, Roche Diagnostics GmbH, Germany) was performed as previously described [5].

### Mitochondrial membrane potential

A total of 4.0×10^5^ HCS-2/8 cells were cultured in each well and treated with Dexa (25 µM) for 12, 24 or 48 hrs. After treatment with Dexa, cells were collected by trypsinization, washed with PBS, resuspended in DMEM-F12 medium containing tetramethylrhodamine ethyl ester (TMRE, 100 nmol) and incubated at 37°C for 60 min. Cell suspensions were then subjected to analyses using an ELISA reader (450 nm absorbance, 570 nm reference) to detect any dissipation of the mitochondrial membrane potential.

### Cytochrome c release

HCS cells were fractionated into cytosolic and mitochondrial fractions and analyzed for the release of cytochrome c into the cytosol using the cytochrome c ELISA kit (R&D systems) according to the instructions provided by the manufacturer. Briefly, cells (2×10^6^) were treated with Dexa (25 µM) and IGF-1 (100 ng/ml) for different time periods and collected by centrifugation. After centrifugation, cells were washed with 1× DPBS and lysed in cell lysis buffer (10 mM HEPES (pH 7.4), 250 mM mannitol, 10 mM KCl, 5 mM MgCl2, 1 mM EGTA) containing 1 mM PMSF and a mixture of protease inhibitors (Roche Molecular Biochemicals). The cells were homogenized in lysis buffer with 30 strokes of a Dounce homogenizer using a B-type pestle. The homogenates were centrifuged at 800× g for 10 min to remove nuclei and cell debris. The resulting supernatant was collected in a new Eppendorf tube and centrifuged at 14,000× g for 30 min at 4°C, which yielded cytosolic supernatant fractions and mitochondria-containing pellets that were analyzed for the presence of cytochrome c.

### Small-interfering RNAs

Human HCS-2/8 chondrocytic cells were transfected with a double-stranded siRNA oligonucleotide designed to interfere with the expression of human Bax (Santa Cruz Biotechnology). A scrambled, non-specific siRNA was used as a negative control. Cells were cultured in DMEM-F12 medium supplemented with 20% FBS 24 hr prior to the transfections. On day 2 of culture, siRNAs were added and the cells were then cultured for another 2 days (48 hrs) in DMEM-F12 with 1% FBS. After 48 hrs of transfection, cells were treated with Dexa for 72 hrs and the amount of cell death was determined using the cell death detection ELISA kit (see above). Gene silencing was confirmed by western blotting.

### Metatarsal organ cultures

Three middle metatarsal bone rudiments were dissected out from the paws of day E20 rat embryos and cultured as previously described [43]. All experiments were approved by the local ethical committee at Karolinska Institutet.

### TUNEL and BrdU incorporation assay

Apoptotic cells were identified by terminal deoxynucleotidyl transferase (TdT)-mediated deoxy-UTP nick end labeling (TUNEL) by using a DNA fragmentation kit (Oncogene Research, Boston, MA). Growth plate chondrocyte proliferation was analyzed by a BrdU cell proliferation kit (Amersham Biosciences, Buckinghamshire, UK) and digital automatic cell counting was performed as previously described [44].

### Flourescence microscopy of cells

Staining of cytochrome c and Bax in HCS cells was performed using anti-cytochrome c antibody or anti-Bax antibody (clone 6A7) (BD Pharmingen, Palo Alto, CA). HCS cells (1.0×10^5^) were grown on glass coverslips and allowed to attach by overnight incubation at 37°C. The next day, cells were exposed to Dexa or IGF-1 for the indicated times. Cells were washed with PBS, fixed in 4% paraformaldehyde for 15 min at 4°C, permeabilized with 0.1% Triton X-100 for 15 min, and washed with 1× TBST. After washing (1× PBS), cells were blocked with BSA in PBS for 1 hr. HCS cells were incubated with anti-cytochrome c antibody or anti-Bax antibody (clone 6A7) (BD Pharmingen, Palo Alto, CA), for 1 hr. Cells were washed in 1× TBST and incubated with the corresponding secondary antibodies conjugated with Alexa Fluor 488 (Molecular Probes, Eugene, OR) for 1 hr. Slides were mounted in DAPI-containing mounting vector shield and examined under a fluorescence microscope (a Nikon E800 fitted with an Olympus DP70 camera). Images were processed with the Image-ProPlus image analysis software (Media Cybernetic).

### Rat growth plate studies

Tibia growth plate samples were used from our previously reported *in vivo* study in which 7-week-old male Sprague Dawley rats (B&K Universal, Sollentuna, Sweden) were treated with Dexa (5 mg/kg body weight; s.c.) for 7 days. The control group received vehicle only [45]. Two sections, one from each group, were mounted on each slide so that all samples were treated under the same conditions.

### Flourescence microscopy of metatarsal bones

Fetal rat metatarsal bones were cultured for 12 days with and without Dexa (1 µM) as previously described [43]. Bones were fixed in 4% formalin, and 5 µm-thick sections were mounted on slides. For immunohistochemistry of active Bax (clone 6A7) and c-terminus HSP60 (Santa Cruz Biotechnology, Santa Cruz, CA), antigen retrieval was performed in citrate buffer (pH 6.0) for 15 min at 95–97°C. The sections were incubated with 10% normal serum for 1 hr, followed by primary antibody at 4°C overnight and secondary antibodies conjugated with Alexa 546 (Molecular Probes Inc., Eugene, Oregon, USA) for 1 hr. Slides were counterstained with DAPI for 15 min, and the resulting fluorescent signals detected by fluorescence microscopy (a Nikon E800 fitted with an Olympus DP70 camera).

### Quantitative histology of the growth plates

Histological evaluations were performed as previously described [17], [46]. Tibia and femur sections from all groups were mounted on the same slides and stained with Alcian blue/van Gieson stain to detect any changes in the matrix [47] [48]. Digital images of Alcian Blue- and van Gieson-stained growth plate sections were collected, and all histological measurements were performed within the central two-thirds of the growth plate using the Olympus MicroImage™ software^4.0^ (Olympus Optical Co., Hamburg, Germany). Fifteen measurements were taken from each sample of the growth plate to determine the growth plate height.

### Statistical analysis

All data are presented as mean values ± SEM. Differences between groups were tested using one-way analysis of variance (ANOVA) followed by the Holm-Sidak post-hoc test.

## Supporting Information

Figure S1 Chondrocyte column density (columns per mm growth plate width) in vehicle and Dexa-treated (2 mg/kg body weight) wild type and BaxKO female mice analyzed after 28 days treatment (*n* = 5).
(TIFF)

Figure S2 Dexa induces growth retardation in cultured fetal rat metatarsal bones. The bones were cultured with Dexa (1 µmol/L) for 12 days and longitudinal bone growth was measured as previously reported (36); (***p<0.001, *n* = 7).
(TIF)
