# Supplementary figures and images for: Ablation of the Pro-Apoptotic Protein Bax Protects Mice from Glucocorticoid-Induced Bone Growth Impairment
Source: PLoS One. 2012 Mar 19;7(3):e33168. doi: 10.1371/journal.pone.0033168 (PMC3307731; doi:10.1371/journal.pone.0033168)

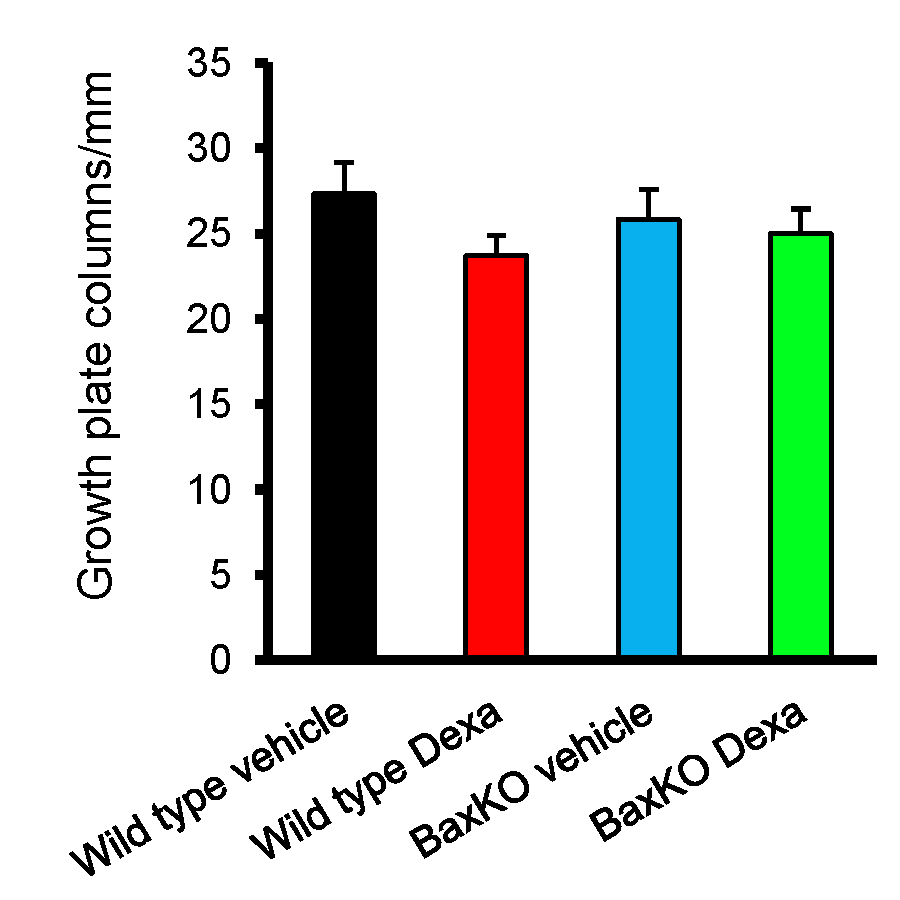

Supplement: Figure S1 — Chondrocyte column density (columns per mm growth plate width) in vehicle and Dexa-treated (2 mg/kg body weight) wild type and BaxKO female mice analyzed after 28 days treatment (n = 5). (TIFF) [file pone.0033168.s001.tif]

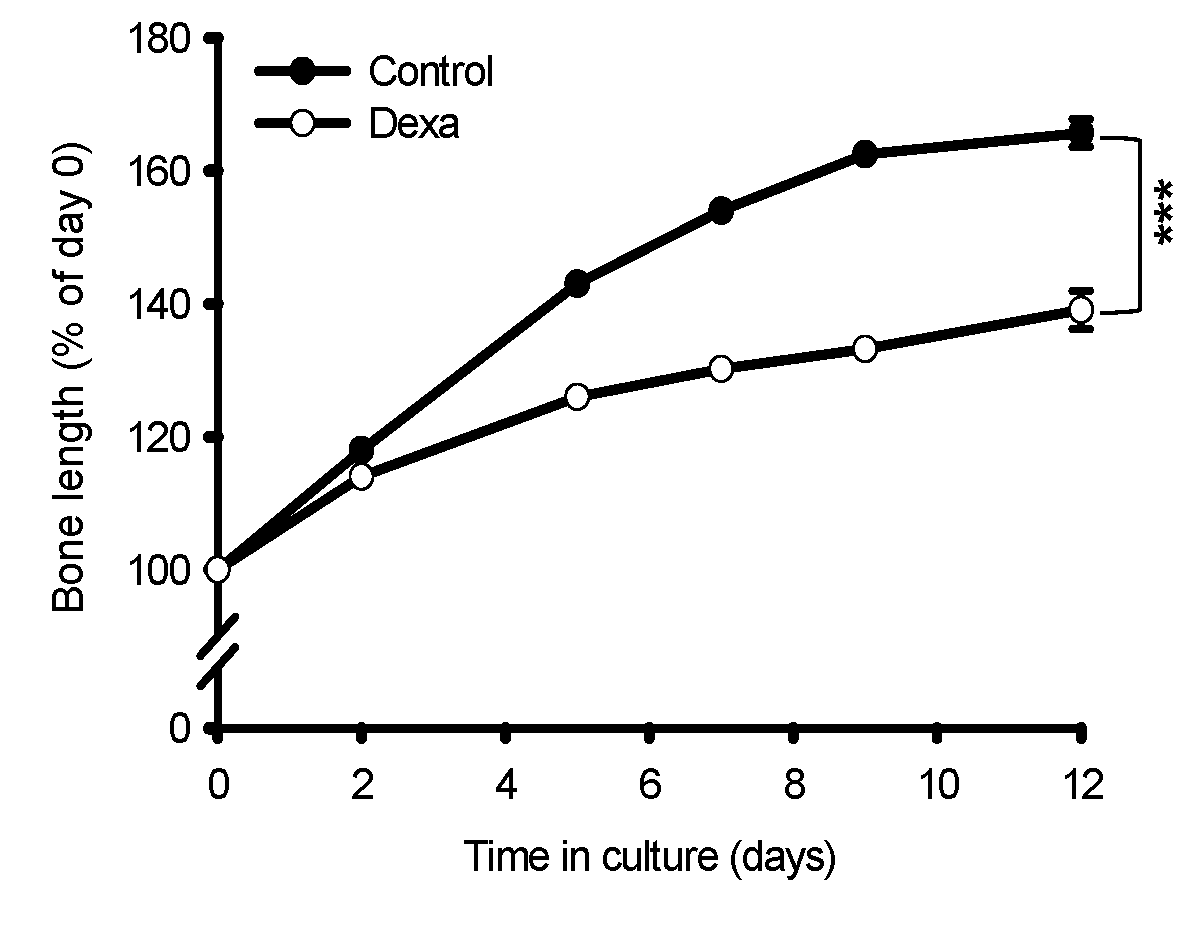

Supplement: Figure S2 — Dexa induces growth retardation in cultured fetal rat metatarsal bones. The bones were cultured with Dexa (1 µmol/L) for 12 days and longitudinal bone growth was measured as previously reported (36); (***p<0.001, n = 7). (TIF) [file pone.0033168.s002.tif]
